# Spectrophotometric assessment of bowel perfusion during low anterior resection: a prospective study

**DOI:** 10.1007/s13304-019-00682-9

**Published:** 2019-10-12

**Authors:** Ibrahim Darwich, Darmadi Rustanto, Ronald Friedberg, Frank Willeke

**Affiliations:** Department of Surgery, St. Marienkrankenhaus Siegen, Kampenstr. 51, 57072 Siegen, Germany

**Keywords:** Bowel perfusion, Low anterior resection, O2C^®^, Colic marginal artery, Anastomotic leak, Cold steel test

## Abstract

Good perfusion of the bowel and a tension-free anastomosis are the two main prerequisites for an uneventful anastomotic healing in rectal surgery. This prospective cohort study investigates the noninvasive intraoperative spectrophotometric assessment of the bowel perfusion using a device called “Oxygen to See” (O2C^®^). Forty patients, planned for low anterior resection, were prospectively enrolled in this study to undergo an intraoperative spectrophotometric assessment of the bowel. Three different O2C^®^ parameters were collected from the colonic and the rectal stumps before fashioning the anastomosis: SO2 (capillary venous oxygen saturation), rHb (relative hemoglobin amount), and flow (blood flow velocity). Bowel perfusion was also assessed with the cold-steel-test (CST), which involves severing the colic marginal artery of Drummond at the tip of the colon stump. The data collected from the spectrophotometric measurement and the CST were analyzed for correlation of both methods with respect to each other and to the outcome of the anastomosis. Nine patients were excluded due to different reasons, thus leaving 31 patients for statistical analysis. Three flow parameters collected at the colonic stump significantly predicted an anastomotic leak (*p*: 0.0057; *p*: 0.0250; *p*: 0.0404). One rHb parameter collected at the rectal stump correlated weakly with the anastomotic outcome (*p*: 0.0768). The CST did not correlate significantly with anastomotic leak (*p*: 0.1195), but showed significant correlations to some rHb values. Intraoperative noninvasive spectrophotometric measurement is feasible and could be a useful method in assessing bowel perfusion before fashioning a colorectal anastomosis.

## Introduction

A symptomatic anastomotic leak (AL) after a low anterior resection (LAR) for rectal cancer is one of the most distressing and feared complications, associated with a high level of morbidity and a leak-related mortality rate of as high as 39% [1–4]. A low rectal anastomosis, neoadjuvant radiation therapy, intraoperative complications, and the male sex have been implicated as independent risk factors for anastomotic leaks [5, 6]. A defunctioning loop ileostomy has been shown to significantly reduce the risk of AL [7]. Yet, while taking those risks into consideration, the surgeon must ensure that the anastomosis is fashioned tension-free and under adequate blood supply to the bowel ends [8].

A high tie (HT) technique, which involves central division of the inferior mesenteric artery (IMA) to achieve maximal bowel length in an LAR [9], has been advocated by many authors [10, 11]. Yet, while other studies suggested that division of the IMA below the origin of the left colic artery (low tie) is preferable in terms of bowel blood supply [12], there is still no clear clinical evidence that favors one technique over the other [13].

Judging blood supply according to the outer appearance of the bowel has been shown to be inadequate for assessing perfusion [14]. As an alternative to assess bowel perfusion, the cold-steel-test (CST) has been described. This involves severing the colic marginal artery of Drummond right at the end of the stump of the descending colon using the scissors and observing the resulting pulsatile jet of blood [15–17].

No other methods played a serious role in evaluating bowel perfusion in colorectal surgery before this study was initiated in 2015.

In this work, we evaluated the intraoperative spectrophotometric assessment of bowel perfusion using a device called “Oxygen to See” (O2C^®^, LEA-Medizintechnik, Giessen, Germany). This device utilizes laser Doppler flowmetry and tissue spectrometry to quantitatively assess tissue perfusion. There is experimental and clinical evidence supporting the reproducibility of measurements of this device [18–21]. There have also been clinical studies describing the use of this device in colorectal surgery. In one study, it was utilized to show a decrease of bowel perfusion after mobilization of the bowel und dividing the mesentery [22]. In another study, it was used to compare the difference in bowel perfusion after performing an HT and a low tie (LT) technique [23]. In both studies, however, the assumption was made that this device was adequate, if not also legitimate, for performing the task of assessing bowel perfusion, yet without supporting evidence. No attempt was made in those studies to define cut-off values of measurements.

We attempted in this study to evaluate the feasibility of intraoperative use of this device and check for its validity by analyzing the correlation of the parameters measured with the CST. Furthermore, we tried to find a correlation between the measured parameters and anastomotic leak.

## Methods

Forty patients, planned for elective laparoscopic or open low anterior resection for rectal cancer, were eligible to be enrolled in this prospective study if they were above 18 years of age and had a Karnofsky index (KI) of 70% or higher. Written informed consent was obtained from all patients. The ethics committee of the Mannheim medical university granted approval for the study protocol (2015-410 M-MA-§23b MPG).

Patients underwent neoadjuvant long-course chemoradiation (nCRT) or short-course radiation therapy alone (nRT) if indicated by our multidisciplinary team (MDT).

All patients received mechanical bowel preparation (MBP) 1 day before surgery unless contraindicated due to bowel stenosis, in which case, MBP was then replaced by an enema on operation day. During this study, oral antibiotics were not given 1 day before surgery, since this was still not standard procedure at our department. All patients received a perioperative intravenous single-dose antibiotic prophylaxis and epidural analgesia.

**Fig. 1:**
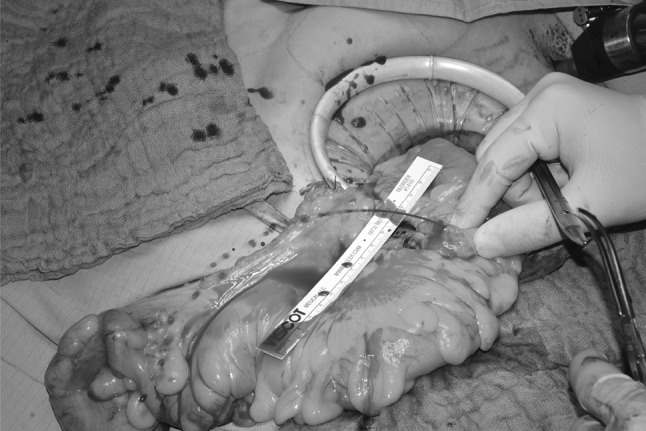
Cold-steel-test performed by severing the colic marginal artery of Drummond. The pulsatile blood jet is measured in centimeters of length

The low anterior resection with total mesorectal excision was done according to the surgical principles defined by Heald et al. and Enker et al. [24, 25]. A high tie technique, defined by division of the IMA at its origin, was performed in all patients. The inferior mesenteric vein was divided below the pancreas and the lesser sac was entered via the medial approach. Dissection in the mesocolic plane was performed until the origin of the middle colic artery was reached and thus allowing for maximal mobilization of the left colon as well as the left-sided transverse colon. Total mesorectal excision und transection of the rectum followed. In a laparoscopic procedure, the specimen was then exteriorized via a small incision in the left middle abdomen allowing for O2C^®^ measurement at the level of the left colon as well as the mobilized transverse colon. Once the mesentery was completely divided and the descending colon transected with a linear cutting stapler machine, a CST was performed after securing a systolic blood pressure of at least 120 mmHg. The length of the resulting pulsatile blood jet was measured in terms of length in centimeters and video documented (Fig. 1). The blood pressure during the CST was recorded.

**Fig. 2:**
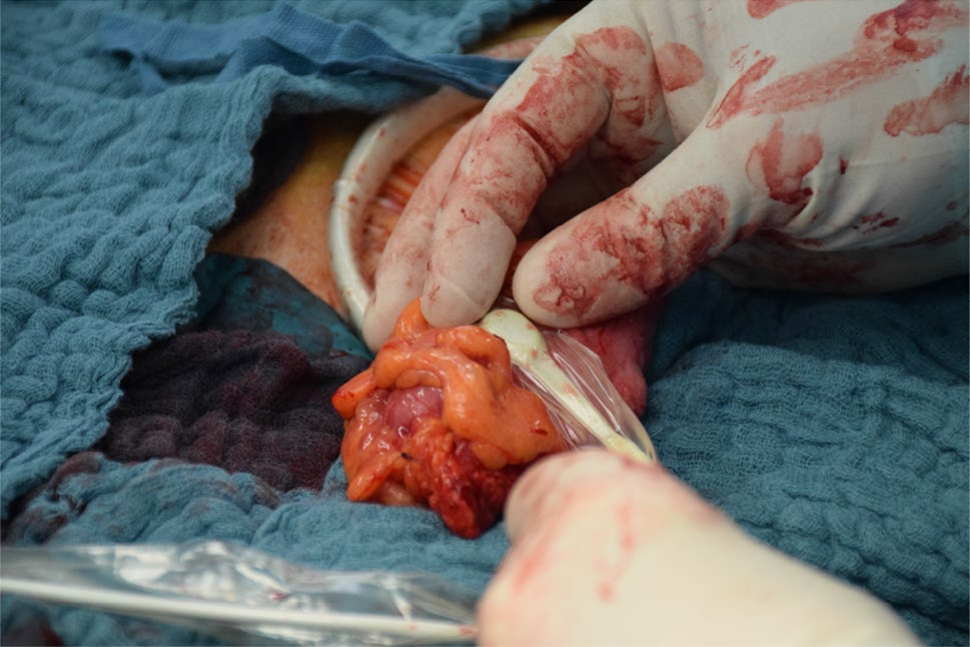
O2C measurement at the colon stump

A flat glass fiber O2C^®^ probe (LFX-55), covered with a sterile polyurethane drape and utilizing laser Doppler flowmetry as well as tissue spectrometry, was used to measure three different parameters (Fig. 2): SO2 (capillary venous oxygen saturation), expressed in percent and reflecting the condition of local tissue hypoxia, rHb (relative hemoglobin amount), expressed in arbitrary units and reflecting venous filling of microvessels, and flow (blood flow velocity), also expressed in arbitrary units and reflecting blood flow velocity.

A digital protocol with designated points of measurement on the colonic and rectal stumps was specifically developed for the study and embedded into the machine’s software. The measurement was performed under ambient light and after turning off the surgical light. The O2C^®^ probe was controlled by a footswitch. The surgeon had a live monitor view throughout the course of measurement at each point of probe placement and could decide anytime to cancel or to save the measured values. Once saved, the parameters could not be manipulated or changed.

**Fig. 3:**
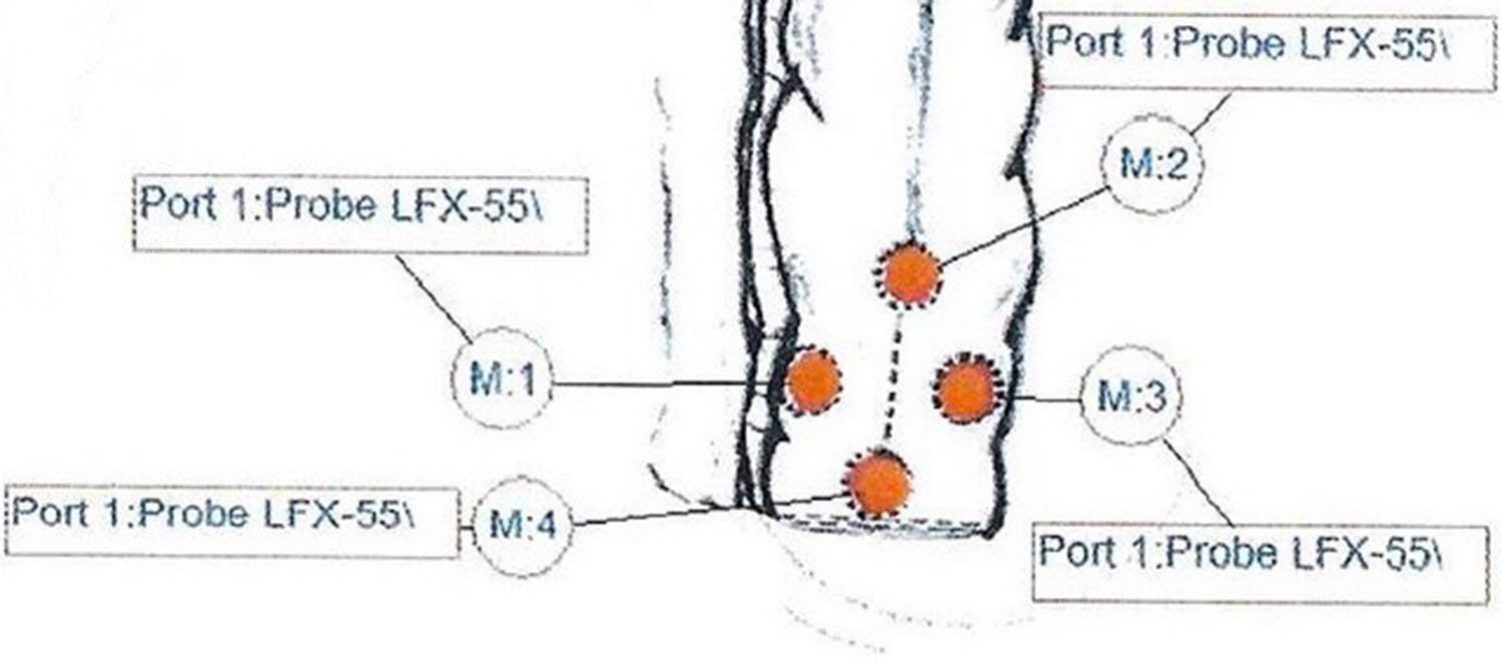
Points of probe placement

**Fig. 4:**
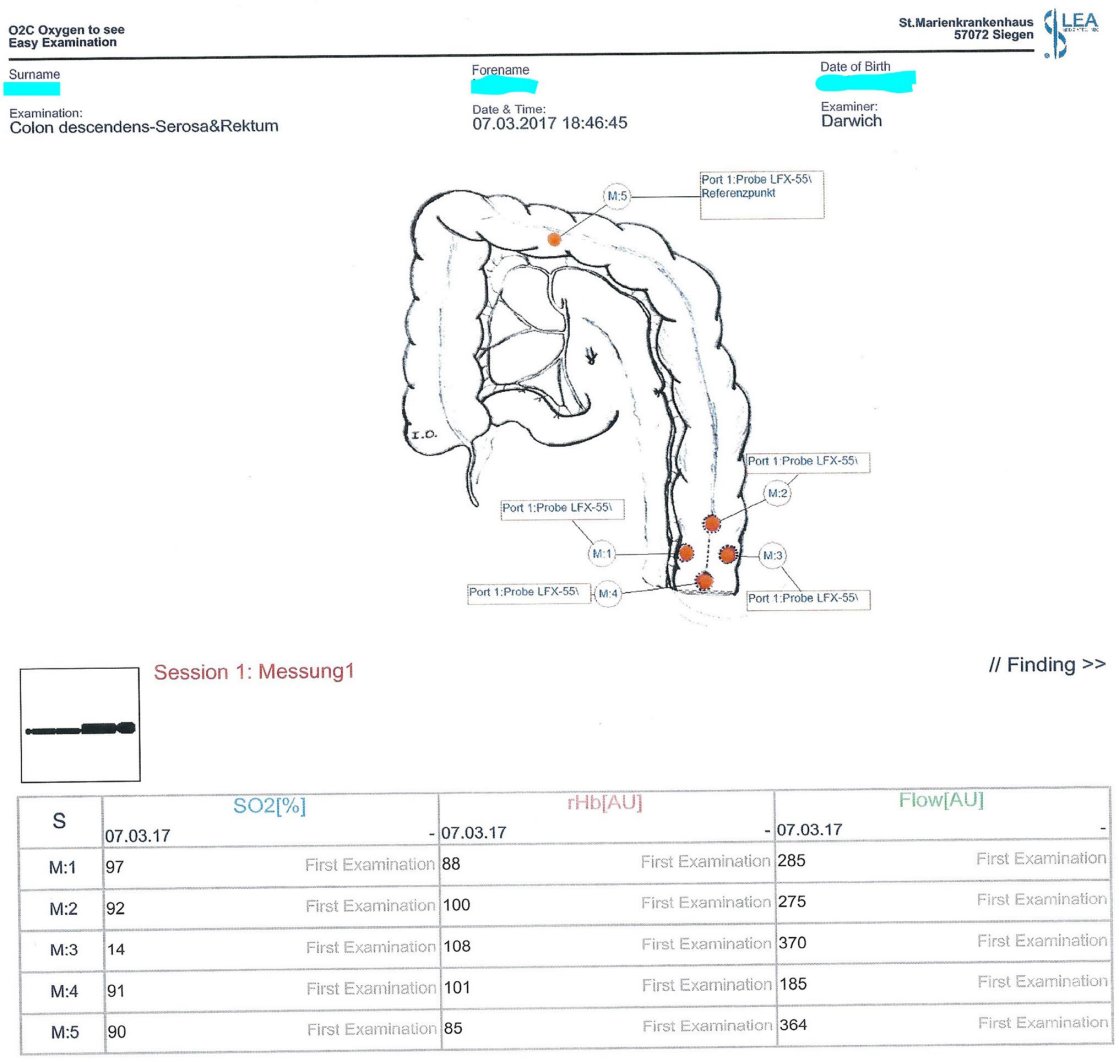
O2C^®^ measurement protocol showing the designated points of probe placement on the colon serosa. M1: mesenteric, M3: antimesenteric, and M2 and M4: proximal and distal, respectively, to the future site of anastomosis. M5 is reference point measured at the transverse colon

The O2C^®^ measurement was then performed according to the aforementioned protocol at four different points on the serosal part of the descending colon stump (M1: mesenteric side, 1 cm proximal to the staple line, M2: central, 3 cm proximal to the staple line, M3: antimesenteric, 1 cm proximal to the staple line, and M4: central and adjacent to the staple line) and at an additional point of reference on the midst of the transverse colon (Figs. 3, 4).

**Fig. 5:**
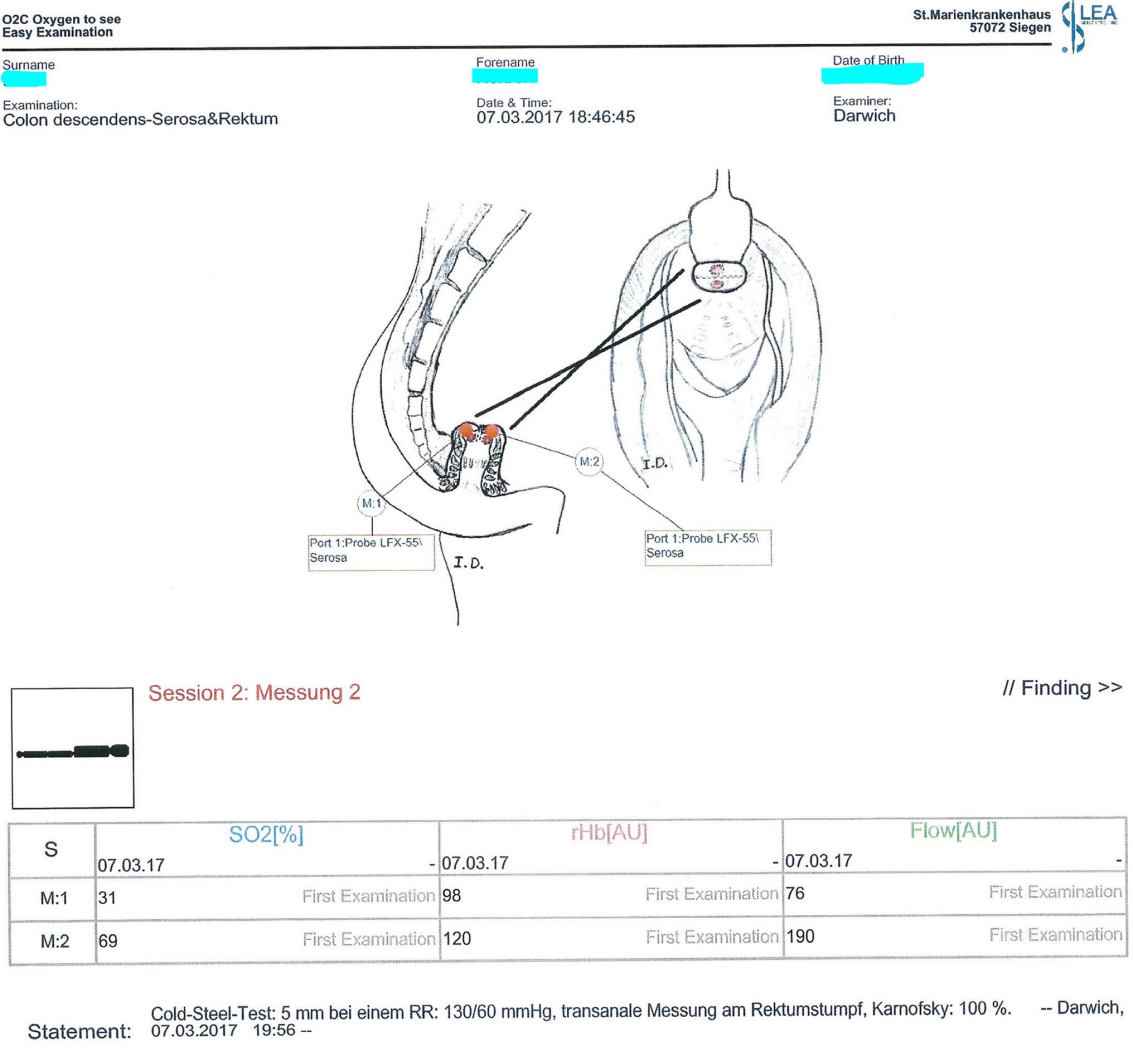
O2C^®^ measurement protocol showing the designated points of transanal probe placement on the rectum mucosa. M1 (Rec1): dorsal and M2 (Rec2): ventral

Following transection of the rectum, O2C^®^ measurement was performed transanally at the rectal stump (M1: dorsal to the staple line and M2: ventral to the staple line) due to better reachability (Fig. 5). If an intersphincteric resection was performed, followed by a hand-sewn coloanal anastomosis, no transanal O2C^®^ measurement was undertaken, since no rectal mucosa was left. One surgeon performed all O2C^®^ measurements.

A side-to-end double-stapled anastomosis was used unless a coloanal hand-sewn anastomosis was fashioned, in which case, an end-to-end reconstruction was chosen. A defunctioning ileostomy was fashioned in all patients unless the surgeon decided to refrain from defunctioning at his or her preference.

All patients received a water-soluble contrast enema during follow-up to assess the integrity of the anastomosis. This was done in case of clinical suspicion of an anastomotic leak during inpatient stay or before closure of the ileostomy (6 weeks–6 months after discharge depending on whether the patient was to receive adjuvant therapy or not). Otherwise and in case no ileostomy was fashioned, a contrast enema was done empirically before discharge to satisfy the study objectives. The results of the contrast enemas were recorded. This constituted the closing-up step of data collection per patient. Anastomotic leakage was defined in this study as a symptomatic leakage leading to therapeutic intervention, with or without re-laparotomy, i.e., grade B and C according to the classification of the International Study Groups of Rectal Cancer (ISREC) [26, 27]. Sorting out ischemic events leading to anastomotic failure was considered paramount in this study to enable a differentiated statistical evaluation. A grade A anastomotic finding requires no change in patient management and heals spontaneously in this regard [28]. This led to the decision not to list a potential grade A finding under anastomotic failure in the study design.

### Statistical analysis

The anastomotic outcome was converted into a binary during statistical analysis (0 for AL and I for no AL). The mean of the data collected per patient at the four points of measurement on the colonic stump and the two points of measurement on the rectal stump was calculated for each of the different O2C^®^ parameters and designated as Mm. Furthermore, the mean and standard deviation of each of the O2C^®^ parameters was calculated for the patient group that had an anastomotic leak and the patient group with an intact anastomosis. Fisher’s exact test, *t* test, Cochran–Armitage test, and Mann–Whitney *U* test were used to check for significance of associations. Correlations between O2C^®^ measurements and the CST were analyzed using correlation coefficients (Pearson and Spearman) to check for validity. Correlations between O2C^®^ data and the anastomotic leak were analyzed with logistic regression. Statistical analysis was conducted with SAS.

Feasibility of intraoperative use and correlation of collected O2C^®^ parameters with the CST constituted the primary endpoint, whereas correlation of the parameters with the anastomotic outcome constituted the secondary endpoint of this study.

## Results

Between 2015 and 2017, 40 patients were enrolled in this prospective study (19 f, 21 m). 61.8% of the patients received neoadjuvant therapy. A diverting ileostomy was performed in 85.3% of the patients who received an anastomosis. A Hartmann procedure had to be done in five of the patients. Three patients did not receive O2C^®^ measurement because of a broken O2C^®^ probe. One patient died on the first day after surgery due to a sudden cardiac arrest (confirmed through autopsy). Thirty-one patients (16 f, 15 m) were left for statistical analysis (Table 1).

**Table 1:** Patient’s characteristics

Variable	Value
Mean age (years)	66.74 (37–89)
Sex (F/M)	16/15
ASA	I (1), II (18), III (12)
KI 70% (*n*)	4
KI 80% (*n*)	2
KI 90% (*n*)	4
KI 100% (*n*)	21

*ASA* American Society of Anesthesiologists Status, *KI* Karnofsky index

The overall rate of anastomotic leakage was 26%, including the three patients who did not undergo O2C^®^ measurement due to probe defect, yet received an anastomosis. Nine patients developed a grade B or grade C anastomotic leakage. Five patients had grade A anastomotic findings, which healed spontaneously on follow-up, so that uneventful ileostomy closure was performed.

Intraoperative O2C^®^ measurement was done in a matter of 3–4 min per patient with no difficulties. In three patients, no measurement was done because of a fractured probe cable, which was then replaced by the manufacturer.

Sex (*p* = 0.2524), age (*p* = 0.3940), neoadjuvant therapy (*p* = 0.4267), performing a defunctioning loop ileostomy (*p* = 0.2933) and surgical technique (laparoscopic vs. open, *p* = 0.3940), were not significant predictors of an increased risk of AL in this study. A reduced Karnofsky index was found on the other hand to significantly increase the risk of AL (*p* = 0.0093). No statistically significant association was found between the length of the pulsatile blood jet during the CST and anastomotic leak (*p* = 0.1195).

The intraoperative systolic blood pressure recorded during the CST had no statistically significant effect on the anastomotic outcome (*p* = 0.7471) (Table 2).

**Table 2:** Analysis of patient characteristics and other variables with respect to anastomotic leak (31 patients fit for analysis)

Variable	Intact anastomosis	Anastomotic leak	Test	*p*
Sex				
Male *n* (%)	9 (60%)	6 (40%)	Fisher’s exact test	0.2524
Female *n* (%)	13 (81%)	3 (19%)		
Mean age				
65 years (*n*)	22		*t* test	0.3940
70 years (*n*)		9		
Technique				
Open *n* (%)	4 (57%)	3 (43%)	Fisher’s exact test	0.3841
Laparoscopic *n* (%)	18 (75%)	6 (25%)		
Karnofsky index				
70%	0 (0%)	4 (100%)	Cochran–Armitage trend test	**0.0093**
80%	2 (100%)	0 (100%)		
90%	3 (75%)	1 (25%)		
100%	17 (81%)	4 (19%)		
Mean CST blood jet				
10.4 cm (SD: 15.27) (*n*)		9	*U* test	0.1195
16.9 cm (SD: 15.42) (*n*)	22			
Mean systolic BP				
133 mmHg (SD: 15.6) (*n*)		9	*t* test	0.7471
135 mmHg (SD: 17.4) (*n*)	22			
Neoadjuvant therapy				
Primary surgery *n* (%)	10 (83.3%)	2 (16.7%)	Fisher’s exact test	0.4184
nCRT/nRT *n* (%)	12 (63.2%)	7 (36.8%)		
Defunctioning ileostomy				
Without ileostomy (*n*)	4 (100%)	0 (0%)	Fisher’s exact test	0.2952
With ileostomy (*n*)	18 (66.7%)	9 (33.3%)		

*CST* cold steel test, *BP* blood pressure, *n* number of patients, *SD* standard deviation

Colonic SO2-M3, Flow-M1, Flow-M3, and the mean flow per patient (Flow-Mm) significantly predicted the risk of an AL in this study (Table 3). A weak association was detected in one other parameter collected at the colonic stump (rHb-M2, *p* = 0.0757). Only one parameter collected dorsally on the rectum stump (rHb-Rec1) showed a weak association with the anastomotic outcome in terms of leakage (*p* = 0.0768). Analysis of the remaining O2C^®^ parameters did not show a statistically significant association with the anastomotic outcome. The mean values of all O2C^®^ parameters collected at the reference point (M5) did not significantly differ between the groups, with and without anastomotic leakage.

**Table 3:** O2C^®^ parameters significantly associated with anastomotic outcome

Variable	Intact anastomosis	Anastomotic leak	Test	*p*
Mean SO2-M3				
88% (SD 17.5) (n)	22		*U* test	**0.0404**
73% (SD 26.0) (*n*)		9		
Mean Flow-M1				
248 AU (SD 109.9) (*n*)	22		*U* test	**0.0250**
163 AU (SD 114.4) (*n*)		9		
Mean Flow-M3				
218 AU (SD 81.3) (*n*)	22		*U* test	**0.0057**
130 AU (SD 67.6) (*n*)		9		
Mean Flow-Mm				
217 AU (SD 55.0) (*n*)	22		*U* test	**0.0177**
162 AU (SD 91.6) (*n*)		9		

*AU* arbitrary units, *n* number of patients, *SD* standard deviation

The above variables that showed a statistically significant as well as a weak association with the anastomotic outcome were then analyzed with logistic regression. This yielded three parameters with varying significance. Flow-M3 was significant (*p* = 0.0348) with an area under the curve (AUC) of 0.881. Flow-Mm and Flow-M1 showed a weak association (*p* = 0.0604, AUC: 0.778 and *p* = 0.0752, AUC: 0.763, respectively).

Statistical analysis looking for associations between the variables (Age, Sex, Karnofsky index, surgical technique, systolic blood pressure, and O2C^®^ parameters) and the length of the pulsatile blood jet during the CST showed a few significant correlations. Sex correlated significantly with the CST (*p* = 0.0160). Furthermore, significant reversed correlations were detected between the CST and rHb-M2 (*p* = 0.0064, *r*: − 0.4787), rHb-Mm (*p* = 0.0138, *r*: − 0.4379), rHb-M4 (*p* = 0.0488, *r*: − 0.3568), and rHb-M3 (*p* = 0.0603, *r* = − 0.3412)^1^

Logistic regression analysis was then used to identify cut-off values for the three flow parameters that predicted a risk of AL. The cut-off values are listed in Table 4 with the sensitivity and specificity for each value. The sensitivity displays the odds of correctly predicting an AL. The specificity displays the odds of correctly foretelling an uneventful anastomotic outcome.

**Table 4:** Cut-off values for O2C^®^ parameters

Variable	Cut-off (AU)	Sensitivity %	Specificity %	AUC	*p*
Flow_M1	208.02	88.89	72.72	0.763	0.0752
Flow_Mm	180.04	88.89	68.18	0.778	0.0604
Flow_M3	164.43	88.89	63.64	**0.823**	**0.0268**

*AU* arbitrary units, *AUC* area under the curve

## Discussion

AL remains one of the most dreaded complications to be dealt with after a low anterior resection. A reliable device for quantitative measurement of tissue perfusion that could predict anastomotic healing after a low anterior resection is still lacking.

This prospective study addresses a device called O2C^®^ that utilizes noninvasive laser Doppler flowmetry and tissue spectrometry for the quantitative assessment of bowel perfusion. This device had previously found colorectal uses in clinical trials, yet only under the eminence based assumption that it is effective in assessing bowel perfusion and thus adequate for investigating other study goals [22, 23].

We looked in this study at three main aspects of this device: feasibility and practicability of the device for intraoperative use, validity of the collected data, and correlation of the collected data with anastomotic leak.

### Feasibility and practicability

The intraoperative use of O2C^®^ proved to be easy and feasible with a minimal time cost of 3–4 min per measurement. Yet, the measuring probe of the device was shown to be sensitive to frequent handling, which resulted in a fractured probe cable and failed measurement in three of the patients recruited for the study.

### Validity of the collected data

In an effort to validate this method of perfusion assessment of the bowel, we analyzed it in comparison to the only other method of assessment available at the time for this purpose, namely the CST. In a prospective study by Novell et al. (Br J Surg 1990; 77: 137–8), it was shown that fashioning an anastomosis despite an absent pulsatile blood flow after transecting the marginal artery at the stump of the left colon significantly increased the rate of anastomotic leak. In an effort to try to utilize the CST as a reference method of validation in our study, we analyzed the association of the length of the pulsatile blood jet in centimeters with the anastomotic outcome. For obvious reasons, anastomoses in this study were fashioned only if pulsatile blood flow out of the marginal colonic artery was confirmed. Furthermore, the association between the CST and different variables, including the measured parameters, was statistically analyzed with the help of correlation coefficients.

While significant reversed correlations were found between the CST and some rHb values measured with the O2C^®^ device, no statistically significant association was found between the length in centimeters of the pulsatile blood jet during the CST and anastomotic leak (*p* = 0.1195) despite a quite interesting observed tendency towards a difference between the groups (10.4 cm in the AL group vs. 16.9 cm in the group with intact anastomoses). This result challenges of course the rationale of validating the O2C^®^ measurements with the CST in this study. Yet, one has to emphasize that the CST was analyzed here in terms of the length of the pulsatile blood jet and not in terms of the absence or presence of pulsatile blood flow in its correlation to anastomotic leak. Thus, it can be suggested that a higher number of cases would have been necessary to achieve statistical significance. A larger study ought to elaborate more on these observations.

### Prediction of anastomotic outcome

An AL rate of 26% in this study was a major concern for the surgeon and a heavy burden for the patients. Yet, this may have enabled a more differentiated statistical evaluation of the O2C^®^ data collected. The causes behind this leak rate may have been diverse. All four patients who had a KI of 70% had an AL. This was also evident in the significant association between the KI and AL (*p* = 0.0093). It is also worthy to mention that at the time, data for this study were collected, administering oral antibiotics 1 day prior to surgery was not standard procedure at our department [29]. Since then, oral antibiotics were added to our regimen of MBP 1 day prior to rectal surgery and we have witnessed a drastic reduction in our AL rate [30].

We found significant associations between O2C^®^ parameters and the anastomotic outcome. The dominating parameter in this respect was the flow parameter, Flow-M3, collected at the antimesenteric side of the descending colon stump (Fig. 5), which predicted AL with high statistical significance (*p* = 0.0057). The lower the value of this parameter, measured before completion of the anastomosis, the higher was the chance of an anastomotic leak. Similar significant associations, although to a lesser extent, were found at Flow-Mm, Flow-M1, and SO2-M3 with *p* = 0.0177, *p* = 0.0250, and *p* = 0.0404, respectively. It was quite surprising to see that data collected at the rectal stump did not associate significantly with anastomotic leakage (with the exception of a weak rHb-Rec1 association collected dorsally and which could not be confirmed by logistic regression analysis). Previous evidence in literature suggested a role of reduced perfusion in the rectal stump in increasing the rate of anastomotic leak [31, 32]. Limited statistical power might have been the reason for not being able to detect an association in our study in this regard.

All the O2C^®^ parameters (Flow, SO2 and rHb), collected at the reference point in the middle of the transverse colon M5 (Fig. 1), did not significantly influence the anastomotic outcome in this study. Bearing in mind that the superior mesenteric artery provides the main blood supply of the transverse colon via the middle colic artery, no associations between the anastomotic outcome and the O2C^®^ parameters, collected at the reference point M5, had been expected. This observation supports the presumption that the measuring process was done evenly and similarly in all patients and that the detected significant associations at the level of the colonic stump were not the result of a handling or user error.

### O2C^®^ cut-off values

Logistic regression analysis yielded three parameters with varying significance: Flow-M3 (*p* = 0.0348, AUC: 0.881), Flow-Mm (*p* = 0.0604, AUC: 0.778), and Flow-M1 (*p* = 0.0752, AUC: 0.763). Also with the help of logistic regression, cut-off values for the above parameters with the highest respective sensitivity and specificity were determined. The O2C^®^ parameter with the highest predicting accuracy of anastomotic outcome was found to be Flow-M3 with a cut-off value of 164.43 AU, a sensitivity of 88.89%, and a specificity of 63.64% (*p* = 0.0268, AUC: 0.823). This means that an anastomotic leak is in 88.89% of the cases likely to be correctly predicted if the Flow-M3 values measured were lower than 164.43 AU.

### Association of O2C^®^ parameters with risk factors of al

Gender, age, neoadjuvant therapy, performing a defunctioning loop ileostomy, and surgical technique were not found to significantly influence the anastomotic outcome in this study. Although a tendency towards more anastomotic leaks in male patients could be observed, it did not reach statistical significance, however. Limited statistical power might have also been an issue here.

A reduced Karnofsky index was found to significantly increase the risk of developing an AL. This parallels data in the literature that associates a high ASA score (American Society of Anaesthesiologists Risk Score) with a higher risk of anastomotic failure [33].

### Comparing O2C^®^ with fluorescence angiography

It is quite noteworthy to state that within the time during which data were collected for this study, several publications appeared describing the use of fluorescence angiography via intravenous injection of indocyanine green (ICG) to assess bowel perfusion [33–36]. Despite the impressive imagery provided by this method showing demarcated green fluorescent perfused regions of the bowel, there is still place here in our opinion for subjectivity of judgment when it comes to surgical decision-making. This is especially evident when the level of fluorescence, observed by the surgeon, is graded as absent, sufficient, and optimal, as described in one of the published papers [35]. This comes in contrast to O2C^®^ which attempts to define objective quantitative measurements in identifying adequate bowel perfusion.

The invasive nature of fluorescence angiography on the other hand, which involves the intravenous administration of ICG, regardless of how low the complication rate is, must also be taken into consideration [37].

Last but not least, cost effectiveness plays a major role during decision-making each time which a healthcare provider considers acquiring new medical technologies. The acquisition costs of the O2C^®^ device at our department amounted to about € 25 000. This comes in contrast to costs of about € 110 000 to acquire near-infrared fluorescence imaging technology [36]. Furthermore, a single-use sterile polyurethane drape, used to cover the glass fiber O2C^®^ probe during intraoperative measurement, costs € 6, whereas ICG costs about € 50 per 25-mg vial [36].

Obviously, comparative studies between the two mentioned techniques are needed to clarify the above raised questions.

### Limitations of this study and future implications

This study has a number of limitations. First, the relatively small number of patients may have resulted in limited statistical power. Second, the prospective, non-randomized, and observatory nature of this cohort study prevented change of surgical strategy in accordance with the collected measurements. The level of transection of the proximal colon was thus based only on the pulsatile flow of the severed marginal artery. Furthermore, the non-randomized structure of this study may have allowed for bias in terms of surgical decision-making as can been seen with the surgeon’s preference not to divert with a loop ileostomy in some patients. Third, a reliable method of testing adequate bowel perfusion other than the “cold-steel-test” to validate the collected O2C^®^ data was not available at the start of this study. This paves the way, however, for a study comparing fluorescence angiography with O2C^®^.

At our surgical department, a further prospective study analyzing O2C^®^ measurements prior to performing lower limb amputations is already underway. Furthermore, a comparative randomized study between O2C^®^ and fluorescence angiography while utilizing the above-mentioned cut-off values is currently being considered.

## Conclusion

Intraoperative O2C^®^ assessment of bowel blood supply is feasible and could be an important tool of a quantitative and noninvasive measurement. The parameters suggested by this study to predict anastomotic leakage could set the basis for further trials in trying to check for their validity. Clearly, high-powered randomized trials are needed.

## Abbreviations

AL: Anastomotic leakage
AUC: Area under the curve
CST: Cold steel test
HT: High tie
ICG: Indocyanin green
IMA: Inferior mesenteric artery
KI: Karnofsky index
LAR: Low anterior resection
MBP: Mechanical bowel preparation
MDT: Multidisciplinary team
nCRT: Neoadjuvant chemoradiotherapy
nRT: Neoadjuvant radiotherapy

## Footnotes

1 ^*^*r*: Pearson correlation coefficient.
